# Plasma cannabinoid concentrations and transference during long-term industrial hemp administration in cattle

**DOI:** 10.1093/jas/skaf418

**Published:** 2025-12-08

**Authors:** Bailey R Fritz, Michael D Kleinhenz, Jason J Griffin, Mikaela M Weeder, Alyssa A Nelson, Andrew K Curtis, Geraldine Magnin, Jonathan Ferm, Roman R Ganta, Johann F Coetzee

**Affiliations:** Department of Anatomy & Physiology, College of Veterinary Medicine, Kansas State University, Manhattan, KS 66506; Department of Clinical Sciences, College of Veterinary Medicine, Kansas State University, Manhattan, KS 66506; John C. Pair Horticulture Center, Kansas State University, Haysville, KS 67060; Department of Anatomy & Physiology, College of Veterinary Medicine, Kansas State University, Manhattan, KS 66506; Department of Anatomy & Physiology, College of Veterinary Medicine, Kansas State University, Manhattan, KS 66506; Department of Anatomy & Physiology, College of Veterinary Medicine, Kansas State University, Manhattan, KS 66506; Department of Anatomy & Physiology, College of Veterinary Medicine, Kansas State University, Manhattan, KS 66506; Department of Diagnostic Medicine/Pathobiology, College of Veterinary Medicine, Kansas State University, Manhattan, KS 66506; Department of Diagnostic Medicine/Pathobiology, College of Veterinary Medicine, Kansas State University, Manhattan, KS 66506; Department of Diagnostic Medicine/Pathobiology, College of Veterinary Medicine, Kansas State University, Manhattan, KS 66506; Department of Anatomy & Physiology, College of Veterinary Medicine, Kansas State University, Manhattan, KS 66506

**Keywords:** cannabinoids, cattle, exposure, industrial hemp, plasma, transference

## Abstract

With recent legalization of industrial hemp (IH) production and increased interest in including IH and its byproducts in cattle feed, there is a need to establish the pharmacokinetic profiles of cannabinoids in cattle and guidelines for animal surveillance programs to ensure safety of cattle products entering the food supply. Our group has previously described the pharmacokinetics of cannabidiolic acid (CBDA) and concentrations of other cannabinoids in plasma. In the present study, the plasma cannabinoid concentrations in twelve (12) Holstein steers receiving alfalfa pellet placebo (PLBO), chlortetracycline (CTC) pellets (1.1 mg/kg/d), IH (5.5 mg/kg/d CBDA; HEMP), or a combination of CTC and IH (COMBO) once daily for 63 d were evaluated. Plasma samples were collected every 7 d from day −7 to 77. Eleven cannabinoids were detected above the lower limit of quantification (LLOQ), with the cannabinoid (−)-7-nor-7-carboxy cannabidiol (CBD-7-acid) reaching the highest concentrations. All cannabinoids except CBD-7-acid were below LLOQ by 14 d after final hemp administration. In cattle not receiving IH, CBD-7-acid was detected in multiple steers and timepoints. CBDA was detected in 4 samples (2 steers from each of the PLBO and CTC groups). Both 9-THC and its precursor, THCA, were detected above LLOQ in a singular sample from a steer in the CTC group. These findings suggest that cattle not receiving IH could have detectable concentrations of CBD-7-acid in the blood if cohoused with animals that are exposed to IH. Sample contamination may have been responsible for the detectable 9-THC, THCA, and CBDA concentrations. Based on our results, CBD-7-acid may be a useful tool for screening cattle for IH exposure. However, given the consistent, detectable concentrations in groups not administered IH, a confirmatory test or specific criteria for interpreting CBD-7-acid concentrations would be warranted. These data will help inform decisions regarding surveillance and tolerances for cannabinoid testing in food animals and animal products entering the food supply.

## Graphical abstract

**Graphical Abstract:**
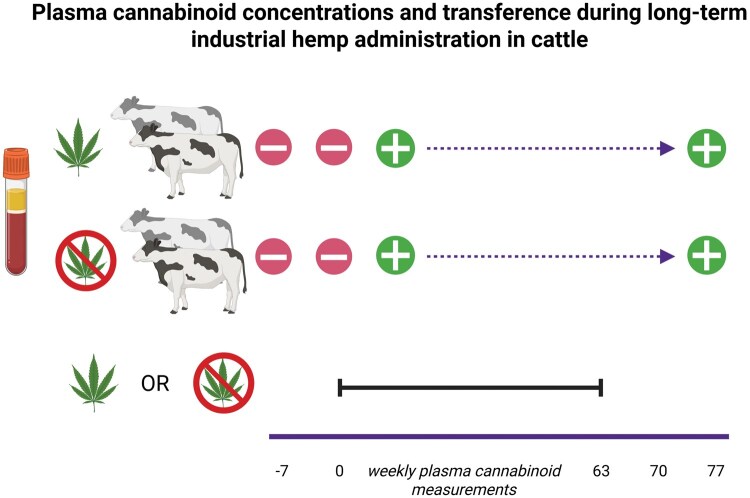

## Teaser

Cattle co-housed with animals that are fed industrial hemp may have detectable CBD-7-acid concentrations in plasma at least 14 d after final exposure. Most cannabinoids are eliminated from plasma within 7 d after final industrial hemp exposure.

## Introduction

Commercial cultivation of industrial hemp (IH; *Cannabis sativa* with ≤0.3% Δ9-tetrahydrocannabinol) was legalized with the 2018 Farm Bill, opening the door for a variety of new markets (Johnson, 2021). The entire IH plant has been leveraged for various products, including fiber, seeds and seed products, and cannabinoid oil extracts (Kleinhenz et al., 2020b). In particular, cannabidiol (CBD) oil extract is a major retail interest in the United States. After extracting CBD (or other cannabinoids) from the cannabinoid-rich flowers and leaves, a large amount of plant material is left behind as “waste.” Because ruminant species, including cattle, are able to digest fibrous plant material, there is increased interest in including IH and its various byproducts in cattle feed. Our group has demonstrated that IH has a favorable digestibility profile and previous work has indicated that various IH products are safe for inclusion in cattle diets (Kleinhenz et al., 2020b; Addo et al., 2023; Smith et al., 2023; Wang et al., 2023; Irawan et al., 2024; Fruge et al., 2025; Irawan et al., 2025; Lopez et al., 2025).

Due to concerns in exposed cattle regarding transfer of cannabinoids into edible products, such as meat or milk, there is a need to establish the pharmacokinetics and tissue depletion profile of various cannabinoids. Our group has previously reported plasma pharmacokinetics and behavioral changes following single and multiple IH doses (Kleinhenz et al., 2020a, 2022). Other groups have published on the depletion of cannabinoids in various tissues (Smith et al., 2023; Fruge et al., 2025; Irawan et al., 2025). Pharmacokinetic parameters can be altered with co-administration of other drugs. Chlortetracycline (CTC) is a commonly used antibiotic in cattle. In humans, it has been reported that the same enzyme is responsible for metabolism of both CBD and tetracycline (Zhao and Ishizaki, 1997; Jiang et al., 2011; Gaston and Friedman, 2017). There is no published information on the concentrations of plasma cannabinoids in cattle when co-administered IH and CTC.

As regulatory agencies investigate potential approval of IH as a feed ingredient, there is a critical need to establish tests for identifying exposed cattle entering the food supply. While methods for tissue matrices are important, analysis of plasma would enable detection of exposed cattle prior to slaughter. Antemortem diagnosis of IH exposure would reduce animal waste by identifying concerning levels of cannabinoid exposure prior to slaughter, allowing producers the time to address any potential violative residues. Currently, there is little research on the long-term plasma depletion of cannabinoids after withdrawal from IH administration, with a single paper evaluating plasma cannabinoid concentrations up to 8 d after withdrawal (Smith et al., 2023). Establishing the time frame for depletion and determining the ideal cannabinoid for use as a surrogate for exposure will assist in regulatory planning of surveillance measures.

The objective of this study was to determine the plasma cannabinoid concentrations in cattle during and after prolonged administration of IH flowers with or without CTC.

## Materials and Methods

### Ethics statement and animal disposition

Experimental procedures were approved by the Institutional Animal Care and Use Committee at Kansas State University (IACUC #4749). All study activities were conducted in conformity to requirements from the United States Department of Agriculture, the State of Kansas, and American Association for Accreditation of Laboratory Animal Care according to *The Guide for the Care and Use of Agricultural Animals in Research and Teaching* (ADSA ASAS PSA, 2020). Six of the steers (receiving either placebo or chlortetracycline—CTC) were sold to a local sale barn following study conclusion; the remaining six steers, which had previously received an experimental ­vaccine, were humanely euthanized via captive bolt and intravenous administration of supersaturated magnesium sulfate in accordance with American Veterinary Medical Association (AVMA) guidelines.

### Animals and housing

Twelve (*n *= 12) Holstein steers 2 yr of age and weighing (±SD) 644 ± 145 kg were enrolled. Steers had been previously acclimated to the research facility and were group-housed in an outdoor dirt pen with access to shelter throughout the study period. Steers were fed a custom grain mix twice daily (86.54% DM, 14.5% CP, 65.91 Mcal/cwt NE_g_, 0.43% calcium, 0.31% phosphorus) at 3% bodyweight per day and had *ad libitum* access to grass hay and water via an automated watering device during the entire study. The pen area supplied per calf (432 ft^2^) exceeded the guidelines established in the Guide for the Care and Use of Agricultural Animals in Research and Teaching for the weight of the steers.

All steers had previously been used in a trial investigating an experimental *Anaplasma marginale* vaccine (IACUC #4643); 6 of the steers received the vaccine, and the remaining 6 were unvaccinated controls. All 12 steers were experimentally infected with *A. marginale* str. St Maries during the previous trial and were confirmed positive on RT-qPCR within 30 d prior to initiation of this study. Due to the biosecurity risks with *A. marginale*, steers were housed in a pen that did not share a fence-line or direct contact with other animals and grass and weeds were mowed within 10 feet of the experimental pens. All steers were treated with acaricide and fly control (UltraBoss Pour-On Insecticide, Merck Animal Health, Rahway, NJ 07065) every 2 wk.

### Experimental design

A restricted randomization technique was applied in order to minimize the number of steers that would have to be euthanized (as steers receiving hemp or the experimental vaccine could not enter the food chain). The 6 experimentally-vaccinated steers were randomized to receive one of the hemp-containing treatments and the 6 unvaccinated steers were randomized to receive one of the treatments without IH. There were 3 steers in each treatment. Treatments included once daily administration of (1) alfalfa pellet placebo (PLBO), (2) chlortetracycline pellets (1.1 mg/kg/d; Aureomycin, Zoetis, Parsippany, NJ 07054; CTC), (3) IH (5.5 mg/kg/d CBDA; HEMP), or (4) combination treatment with CTC and IH as described above (COMBO) for 63 d. The obvious nature of the treatments precluded blinding of the investigators administering treatments. The authors recognize the severe limitations of the treatment allocation, as experimental vaccine status was a confounder with IH treatment. This restricted assignment scheme was chosen out of a desire to minimize unnecessary animal waste, considering that this was pilot data to be used to generate further hypotheses.

### Treatment preparation and administration

Prior to study initiation, the CBDA content as a percentage of total IH weight was determined using ultra-performance liquid chromatography triple quadrupole mass spectrometry (UPLC-MS/MS) and was used to calculate IH doses on an as-fed basis. Cattle in the hemp groups received IH at a dose of 5.5 mg/kg cannabidiolic acid (CBDA) by oral bolus. This dose was based on our previous studies, initially targeting an IH dose of 5 mg/kg CBDA, which was modeled after the 5 mg/kg CBD dose reported for human patients suffering from seizures due to Lennox-Gastaut and Dravet syndromes (Kleinhenz et al., 2020a, 2022). Hemp flower material was finely chopped using a food chopper. Ground IH was placed in gelatin capsules and the weight of the capsules (with empty capsule weight tared) was recorded. Chlortetracycline dosages were calculated weekly based on the updated steer weights and doses were weighed and placed into pre-labeled bags. Aliquots of alfalfa pellets similar to the average weight of chlortetracycline pellets were placed into labeled bags. Alfalfa pellets were chosen for the PLBO treatment as they were the feed that was the most visually similar to IH and CTC and were readily accessible.

Industrial hemp was grown and handled in keeping with licensing requirements under the Kansas Department of Agriculture Industrial Hemp Research Program (license numbers: KDA-0621466839 and KDA-0302873296). The cultivars Endurance HT and CW117 were used (harvested October 2021); cannabinoid content of these cultivars is shown in Table 1. The inflorescence material used in this study was stored at −20 °C until the time of this study (summer 2022) and analysis (spring 2025).

**Table 1.** Plasma cannabinoid concentrations (median; 95% CI) in Holstein steers administered IH inflorescence with or without CTC

	Cannabinoid	Day 7	Day 14	Day 21	Day 28	Day 35	Day 42	Day 49	Day 56	Day 63	Day 70	Day 77	LLOQ, ng/mL
***HEMP***	CBCA	2.6 (0, 6.7)	0 (0, 6.2)	5.4 (1.2, 8.2)	3 (0, 6.6)	3.4 (2.5, 4.6)	2.6 (0, 6.2)	2.5 (0, 5.4)	4.4 (2.0, 7.8)	0 (0, 4.4)	N/D	N/D	2.5
	CBD-7-acid	287 (156, 416)	370 (129, 646)	523 (121, 944)	297 (110, 536)	304 (136, 481)	630 (309, 961)	477 (321, 594)	657 (76, 1,125)	866 (0, 1,866)	601 (0, 1,358)	41 (0, 110)	1
	CBD	1 (0, 2.3)	0 (0, 1.9)	0	N/D	N/D	N/D	N/D	1.4 (0.3, 2.6)	N/D	N/D	N/D	1
	CBDA	92 (0, 290)	131 (0, 309)	154 (36, 304)	53 (33, 75)	123 (32, 197)	83 (29, 138)	70 (41, 98)	113 (0, 356)	52 (1.1, 117)	N/D	N/D	2.5
	CBDVA	2.7 (0, 8.0)	3.6 (0, 9.5)	3.2 (0, 7.8)	2.8 (0, 6.2)	4.9 (0.6, 8.8)	3.5 (0.6, 7.7)	0 (0, 7.2)	4.1 (0, 9.0)	4.3 (1.2, 7.4)	N/D	N/D	2.5
	CBGA	N/D	N/D	N/D	N/D	N/D	N/D	N/D	0 (0, 4.4)	N/D	N/D	N/D	2.5
	CBLA	0 (0, 4.9)	N/D	3 (0, 7.6)	N/D	0 (0, 4.8)	N/D	N/D	3.4 (2.2, 5.2)	N/D	N/D	N/D	2.5
	THCA	6.5 (1.4, 12)	8 (2.2, 13)	6.8 (5.2, 8.8)	4.5 (3.5, 5.8)	7.9 (5.2, 9.6)	5.5 (1.4, 9.9)	4.4 (4.2, 4.6)	7.1 (2.5, 15)	5 (1.8, 9.3)	N/D	N/D	1
	THCV	N/D	0 (0, 1.9)	1.4 (1.3, 1.6)	N/D	N/D	N/D	N/D	N/D	N/D	N/D	N/D	1
	THCVA	N/D	N/D	N/D	N/D	N/D	N/D	N/D	0 (0, 4.6)	0 (0, 4.6)	N/D	N/D	2.5
***COMBO***	CBCA	3 (1.4, 5.5)	4.5 (0.5, 8.0)	3.8 (1.7, 5.9)	2.6 (0, 6.0)	4.3 (2.2, 6.2)	2.9 (1.7, 4.8)	3.1 (1.9, 4.3)	5.1 (2.3, 8.1)	0 (0, 4.6)	N/D	N/D	2.5
	CBD-7-acid	392 (240, 588)	558 (228, 863)	770 (199, 1,224)	435 (148, 650)	466 (0, 1,606)	661 (0, 1,534)	683 (140, 1,047)	902 (180, 1,610)	995 (21, 2,246)	542 (0, 1,702)	157 (0, 296)	1
	CBD	1.1 (0.8, 1.5)	0 (0, 2.7)	N/D	N/D	N/D	0	1.1 (0.6, 1.7)	1.4 (0.7, 2.3)	1.1 (0, 2.5)	N/D	N/D	1
	CBDA	140 (8.9, 237)	141 (87, 199)	146 (0, 282)	43 (20, 68)	78 (45, 100)	92 (49, 122)	80 (31, 137)	155 (0, 393)	44 (0, 156)	N/D	N/D	2.5
	CBDVA	3.3 (0, 9.2)	3.8 (1.0, 6.3)	3.1 (0, 9.7)	0 (0, 7.8)	4.9 (1.7, 7.2)	4.8 (0.6, 7.9)	3.2 (0, 8.2)	3.9 (0.8, 8.8)	6.4 (0.5, 10)	N/D	N/D	2.5
	CBLA	0 (0, 5.8)	3.1 (0, 6.6)	2.7 (0, 5.9)	N/D	2.6 (0, 6.0)	N/D	0 (0, 4.8)	3.8 (0, 9.2)	N/D	N/D	N/D	2.5
	THCA	5.7 (2.5, 11)	9.2 (5.4, 12)	5.6 (1.5, 12)	4.5 (1.1, 8.0)	6.3 (4.5, 9.0)	4.3 (1.7, 7.3)	5.1 (1.1, 9.1)	9.6 (1.7, 17)	4.8 (2.8, 6.4)	N/D	N/D	1
	THCV	N/D	1.1 (0, 3.5)	1.6 (0, 3.7)	N/D	N/D	N/D	N/D	N/D	N/D	N/D	N/D	1
	THCVA	N/D	N/D	N/D	N/D	N/D	N/D	N/D	N/D	0 (0, 5.3)	N/D	N/D	2.5

Plasma cannabinoid concentration summary data for steers in the PLBO and CTC groups can be found in Supplementary Table S2.

Steers received treatments once daily for 63 d. Feed tubs were labeled and assigned to one steer for the entire study to prevent any cross-contamination of treatments. Steers in the PLBO and CTC groups received their treatments loose in their feed tubs with approximately 500 g of textured feed (Producer’s Pride 12% Sweet Textured Livestock Feed, Tractor Supply Co., Brentwood, TN). Steers in the HEMP or COMBO groups were restrained in a chute, a rope halter placed, and the IH was administered via oral bolus; they were then released from the chute and given approximately 500 g of textured feed in their feed tubs. Steers were monitored during feeding to ensure CTC crumbles and IH boluses were consumed. Occasionally, some steers had to receive part of their CTC crumbles in a bolus or the IH capsules were re-administered if not all of the crumbles were consumed or if the boluses were spit out. Following the 63-d treatment period, steers received only their routine diet (grain mix and hay).

### Sample collection

Whole blood samples were collected at −7, 0, 7, 14, 21, 28, 35, 42, 49, 56, 63, 70, and 77 d after initial treatment for complete blood count analysis and cannabinoid concentrations. The 0 d timepoint was collected immediately prior to the first treatment administration. Jugular or coccygeal venipuncture was performed with an 18-gauge 1.5-inch needle and 20 mL syringe or vacutainer system. The blood samples were directly placed into 2 pre-labeled 6 mL K3-EDTA blood tubes, placed on ice, and transferred to the lab. Blood for complete blood count analysis was immediately analyzed using an IDEXX ProCyte Dx hematology analyzer (IDEXX Laboratories, Inc., Westbrook, Maine 04092) and a manual packed cell volume was calculated. Blood smear analysis was performed on any samples exhibiting reticulocytosis or band cells. Blood for cannabinoid analysis was centrifuged at 3,500 relative centrifugal force for 10 min at 4 °C and the plasma was aliquoted into cryovials and frozen at −80 °C for future analysis.

### Animal monitoring

Due to potential for recrudescence of clinical signs due to anaplasmosis, steers underwent a weekly monitoring protocol to screen for signs of disease. This screening included a complete blood count, manual hematocrit, rectal temperature, and observation of behavior. If the red blood cell count or manual hematocrit were below 24%, rectal temperature was >105 °F, or inappetence, standing with legs stretched out, excessive recumbency, or reluctance to rise were observed, the steers were evaluated by a veterinarian and a treatment plan was determined by discussion between study personnel, the veterinarian, and the IACUC attending veterinarian. Assessment of inappetence, stance, and recumbency were based on the subjective observations of one author (B.R.F.) who was familiar with these specific steers; observations were made when interacting with the steers surrounding the time of once daily treatment administration. Steers were also observed once daily during treatment administration (not as part of the weekly IACUC monitoring protocol) and any signs of disease reported to the principal investigator and IACUC attending veterinarian.

### Blood cannabinoid concentrations

Plasma cannabinoids were measured as previously described (Kleinhenz et al., 2020a). Briefly, all solvents used, such as methanol, acetonitrile, isopropanol, and formic acid were LC–MS grade. Individual cannabinoid standards were purchased as solutions in methanol (Cerilliant Corporation, Round Rock, TX), including: (+)-11-nor-9-carboxy-Δ9-tetrahydrocannabinol glucuronide (THC-acid-glu), (−)-11-nor-9-carboxy-Δ9-tetrahydrocannabinol (THC-acid), ( ±)-11-hydroxy-Δ9-tetrahydrocannabinol (THC-11-OH), cannabidivarinic acid (CBDVA), cannabidivarin (CBDV), cannabidiol (CBD), cannabidiolic acid (CBDA), Δ9-tetrahydrocannabinolic acid A (THCA), cannabigerolic acid (CBGA), cannabigerol (CBG), Δ9-tetrahydrocannabinol (9-THC), Δ8-tetrahydrocannabinol (8-THC), cannabichromene (CBC), Δ9-tetrahydrocannabivarin (THCV), cannabichromenic acid (CBCA), and cannabinol (CBN). Cannabinoid analogs used as internal standards included ( ±)-cis-11-nor-9-carboxy-Δ9-tetrahydrocannabinol glucuronide-d_3_ (THC-acid-glu-d_3_), cannabidiol-d_3_ (CBD-d_3_), Δ9-tetrahydrocannabinol-d_3_ (9-THC-d3), ( ±)-11-nor-9-carboxy-Δ9-tetrahydrocannabinol-d_9_ (THC-acid-d_9_), ( ±)-11-hydroxy-Δ9-tetrahydrocannabinol-d_3_ (THC-11-OH-d_3_), and cannabichromene-d_9_ (CBC-d_9_). All cannabinoids standards were stored at − 20 °C.

On the day of analysis, plasma samples were thawed at room temperature. Plasma, internal standard mixture (200 ng/mL), and acetonitrile with 0.1% formic acid were combined to precipitate plasma proteins. Internal standard mixture was not added to the negative controls. Following vortexing and centrifugation, the supernatant was diluted with ultra-pure 18 MΩ·cm water. Samples were loaded onto an Oasis PRIME HLB solid phase extraction plate (Waters Corp., Milford, MA) via a nitrogen positive pressure manifold. Washes were performed with methanol: water (25:75) and elution was performed with acetonitrile: methanol (90:10). Eluates were diluted with water prior to analysis.

Cannabinoid analysis was performed using an Acquity H class UPLC and a TQ-S triple quadrupole mass spectrometer (Waters Corp., Milford, MA). Chromatographic separation was achieved using a UPLC column (100 × 2.1 mm, 1.8 µ, Eclipse Plus C18, Agilent Technologies, Santa Clara, CA) heated at 55 °C. The mobile phase consisted of a gradient of water containing 0.1% formic acid (A) and acetonitrile (B) as follows: 0 min: 60% B, 6.50 min: 86% B, 7.50–9 min: 100% B, 9.01 to 12 min: 60% B. The flow rate was set at 0.5 mL/min, injection volume was 5 µL, and the run time per sample was 12 min. Data acquisition was performed using electrospray ionization in positive and negative mode using multiple reaction monitoring. Linear regression with a weighting factor of 1/X was used and accepted if the coefficient of determination R^2^ was >0.99. Calibration curves were linear from 0.1 to 100 ng/mL for all cannabinoids. The lower limit of quantification (LLOQ) for each detected cannabinoid is presented in Table 1. For cannabinoids not detected or below the LLOQ, which are not presented in Table 1, the LLOQ values were: 1.0 ng/mL for CBC, CBG, CBN, 8-THC, 9-THC, THC-11-OH, and THC-acid-glu; and 2.5 ng/mL for CBD-6-OH, CBD-7-OH, CBDV, and THC-acid. Accuracy and relative standard deviation of the quality control samples can be found in Supplementary Table S1.

Cannabinoid extraction and analysis was performed in batches by one author (G.M.) who was blinded to treatment assignment. Each batch included 20 test samples, in addition to the calibration curve, negative control, and quality control samples. The length of the run time and decreased stability of extracted samples after 12 hours precluded inclusion of more test samples per batch. Use of internal standards enabled accounting for between-batch variation in extraction efficiency and instrument variability.

### Statistics

Cannabinoid concentrations were summarized and analyzed using statistical software (JMP Pro Version 16.0, SAS Institute Inc., Cary, NC, United States). Summary statistics were generated for each cannabinoid within each group and timepoint. Due to the small sample sizes, nonparametric analysis was performed. To analyze the effect of treatment (HEMP vs. COMBO) on cannabinoid concentrations, the Wilcoxon exact test was used. For analysis of time effects, the Friedman rank test was performed. Significance was set *a priori* at *P *≤ 0.05. Figures were made using GraphPad (GraphPad Prism, 10.0, La Jolla, CA, United States).

## Results

One steer in the CTC group had transient neutropenia on day 14 (resolved on recheck complete blood count the following day) and one steer in the COMBO group was noted to be mildly lethargic on day 21. Neither steer required therapeutic intervention. No other adverse events were noted during the study.

Mean cannabinoid concentrations (± SD) of the IH cultivar Endurance HT were: 2.15 ± 0.0096 mg/g 9-THC, 3.23 ± 0.015 mg/g THCA, 1.85 ± 0.013 mg/g CBC, 4.48 ± 0.016 mg/g CBCA, 13.90 ± 0.023 mg/g CBD, 79.88 ± 0.26 mg/g CBDA, and 2.06 ± 0.0026 mg/g CBGA. Mean cannabinoid concentrations of the IH cultivar CW117 were: 1.73 ± 0.0039 mg/g 9-THC, 3.40 ±0.00017 mg/g THCA, 1.77 ± 0.0054 mg/g CBC, 5.05 ± 0.0057 mg/g CBCA, 10.39 ± 0.0060 mg/g CBD, 87.92 ± 0.016 mg/g CBDA, 2.82 ± 0.00028 mg/g CBGA, and 2.68 ± 0.00037 mg/g CBDVA.

**Figure 1.**
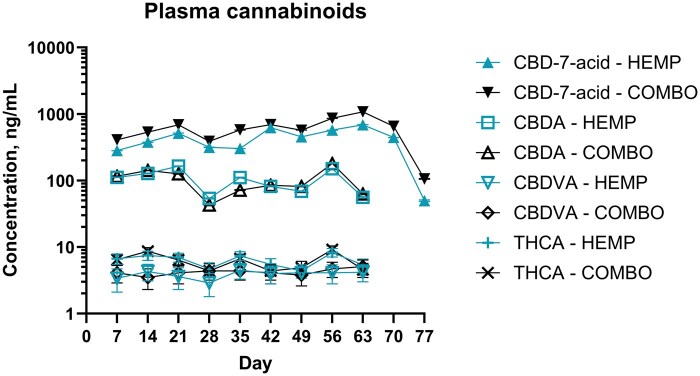
Concentrations of CBD-7-acid, CBDA, CBDVA, and THCA (back transformed logarithmic mean ± SE) in Holstein steers administered IH flowers (HEMP; 5.5 mg/kg/d CBDA) or IH flowers and CTC (COMBO, CTC at 1.1 mg/kg/d) by mouth once daily for 63 d. Sampling occurred every 7 d from day −7 to 77. No cannabinoids were detected on days −7 or 0. Error bars are not shown if smaller than the symbol. Plasma cannabinoid concentrations in steers from the PLBO and CTC groups are presented in Supplementary Figure S1.

No cannabinoids were detected above the LLOQ on days −7 or 0 in any group (see Table 1 and Figure 1 for HEMP and COMBO groups and Supplementary Table S2 and Figure S1 for PLBO and CTC groups; raw cannabinoid data is presented in Supplementary Tables S3–S6.). The cannabinoids CBCA, CBD-7-acid, CBD, CBDA, CBDVA, CBGA, CBLA, 9-THC, THCA, THCV, and THCVA were detected above the LLOQ. There were 3 THCV-positive samples (2 from the HEMP group, 1 from the COMBO group) and 1 CBGA-positive sample (HEMP group). In the HEMP and COMBO groups, only CBD-7-acid was detectable above LLOQ on day 70 and 77. Time did not have a significant effect on concentration for any cannabinoid (*P *> 0.05). There was a significant effect of treatment for CBD-7-acid concentrations (*P *= 0.02, two-tailed), where the COMBO group had higher rank concentrations than HEMP. There was no significant effect of treatment for any other cannabinoid (*P *> 0.05).

In the PLBO group, CBD-7-acid was detected above LLOQ at all timepoints except days −7, 0, and 7 [median: 0 ng/mL (95% CI 0.63 to 4.40); minimum: 0 ng/mL, maximum 32.2 ng/mL]. In the CTC group, CBD-7-acid was detected above LLOQ at all timepoints except days −7, 0, 7, 28, 35, and 49 [median: 0 ng/mL (95% CI 0.31 to 1.24); minimum: 0 ng/mL, maximum: 6.8 ng/mL]. There were a total of 2 CBDA-positive samples each from the CTC (day 56 and 63) and PLBO (day 56) groups (range: 64 to 312 ng/mL). The psychoactive cannabinoid, 9-THC, and its precursor, THCA, were detected in a single steer in the CTC group on day 70 (9-THC: 1.3 ng/mL, THCA: 29 ng/mL). In the CTC and PLBO groups, CBD-7-acid was detectable above LLOQ on days 70 and 77 (range: 1.2 to 3.2 ng/mL).

Due to the lack of IH administration in the PLBO and CTC groups and the infrequent detection of 9-THC, THCA, and CBDA, the differences between measured concentrations and LLOQ were analyzed further. Differences between measured concentrations and LLOQ were calculated using Excel; for observations with undetectable concentrations, the difference was recorded as zero. Using JMP, the Wilcoxon signed rank test was used to assess whether these values were different from zero (i.e. no difference from LLOQ) for each treatment group. A total of 6 comparisons were made: CBD-7-acid and CBDA for both the PLBO and CTC groups and 9-THC and THCA for the CTC group only. A Bonferroni correction was made: significance for these comparisons was set at *P *≤ 0.008, or 0.05÷6. The differences between the CBD-7-acid concentrations and LLOQ in the PLBO and CTC groups were greater than zero (right-tailed *P*-values: ≤0.0001). The difference between measured concentrations and LLOQ was not different than zero for all other comparisons (*P *> 0.008).

## Discussion

This report describes plasma cannabinoids after withdrawal from IH administration, with samples collected 14 d after the last IH dose. A previous study by Smith et al. (2023) evaluating depletion of cannabinoids sampled plasma up to 8 d following the last day of feeding hempseed meal. We detected a total of 11 cannabinoids, including 8 acidic cannabinoids. No cannabinoids were detected on days −7 or 0 in any group. We observed detectable concentrations of the cannabinoids CBD-7-acid 14 d after the final dose of IH was administered, whereas other cannabinoids were not detected by 7 d after the final dose of IH. We did not observe a significant effect of time on cannabinoid concentrations. There was only a significant effect of treatment (comparing HEMP vs. COMBO treatments) for CBD-7-acid. In general, cannabinoid concentrations were relatively static throughout the feeding period, with consistently, numerically lower concentrations on day 28. The cannabinoid CBD-7-acid was detected at low concentrations throughout the feeding period in steers not receiving IH, and CBDA was detected in 4 samples from steers not receiving IH; THCA and 9-THC were detected in a single animal in the CTC group.

The plasma cannabinoid profiles we described in this study are similar to previous reports that observed predominantly acidic cannabinoids in plasma (Kleinhenz et al., 2020a, 2022; Smith et al., 2023; Fruge et al., 2025). Studies evaluating IH flowers and leaves have detected CBCA, CBD-7-acid, CBD, CBDA, CBDVA, CBGA, THCA, and THCVA (Kleinhenz et al., 2020a, 2022; Fruge et al., 2025). In cattle receiving hempseed cake, Smith et al. (2023) reported detectable concentrations of CBCA, CBDA/THCA, CBDVA, and CBNA. Irawan et al. (2025) detected additional, non-acidic cannabinoids, including CBG, 9-THC, and THC-11-OH in cows fed spent hemp biomass (SHB). Compared to these studies, Wagner et al. (2022) detected mostly neutral cannabinoids, including CBD, CBDV, 9-THC, and THCV, along with THCA, in plasma of cows fed IH silage. This suggests that acidic cannabinoids are readily absorbed from the rumen but are eliminated more slowly from the plasma than neutral cannabinoids, potentially due to ion trapping, protein binding, or lower lipophilicity. Irawan et al. (2025) calculated increased absorption rates of CBCA and CBDA compared to their neutral forms and Wagner et al. (2022) reported a much lower milk-to-plasma ratio for THCA compared to 9-THC or the other neutral cannabinoids detected. Similarly, in dogs, CBDA and THCA appear to be more readily absorbed than CBD and 9-THC, respectively (Wakshlag et al., 2020).

Like the cannabinoid profiles we report, the cannabinoid elimination data from this study is consistent with prior literature. Fruge et al. (2025) found that CBD, CBDA, and THCA rapidly depleted to very low plasma concentrations within 96 hours of the last feeding of IH leaves. In a study evaluating hempseed cake, Smith et al. (2023) also reported undetectable plasma CBDA/THCA and CBDVA by day 2 of withdrawal. After a single dose of IH flowers, Kleinhenz et al. (2020a) observed rapid depletion of CBDA within 96 hours, calculating a mean residence time of 30.6 hours. In that study, CBCA and THCA were undetectable after 72 hours and CBDVA concentrations were approximately 1 ng/mL. In the subsequent 14-d feeding study, the authors reported all cannabinoids except CBDA were undetectable after day 2 of withdrawal; CBDA was undetectable on day 5 (Kleinhenz et al., 2022).

In the present study, most of the acidic and neutral cannabinoids were eliminated from the plasma by 7 d after final IH administration (the first blood collection after the last dose). However, CBD-7-acid was detected 7 and 14 d after the final dose. The FDA has a published interest in the inactive metabolite CBD-7-acid, as it reaches concentrations considerably higher than the parent, CBD, and has a much longer elimination half-life of up to 33 hours (FDA CDER, 2017; Taylor et al., 2018; FDA CDER, 2023). In horses, CBD-7-acid elimination is also prolonged, with one study reporting a terminal elimination half-life of 79.85 hours (Eichler et al., 2023). A recent report in cattle indicated that plasma CBD-7-acid did not reach steady state concentrations during a 14-d feeding period and had depleted minimally 5 d after the final dose was administered (Xu and Murphy, 2024). Similarly, Fruge et al. (2025) reported minimal changes in plasma CBD-7-acid in the 96 hours following the end of a 14-d period of IH administration.

Although 9-THC was not detected above the LLOQ in any sample from the HEMP or COMBO groups, one steer in the CTC group had detectable concentrations near the LLOQ (1.3 ng/mL detected vs. 1 ng/mL LLOQ). That same sample had THCA detected at concentrations up to 9 times higher than in the HEMP and COMBO groups (29 ng/mL vs. 3.2 ng/mL, the minimum non-zero value in the HEMP and COMBO groups). The unexpected and sporadic nature of the CBDA, 9-THC, and THCA findings begs the question whether the samples with detectable concentrations we identified were true positives or if they potentially represent sample contamination (e.g. during extraction) or detection of an unknown cannabinoid with the same transition and retention parameters. These cannabinoids were confirmed present based on the qualifier ion. However, statistical analysis revealed these samples were likely outliers, as CBDA, 9-THC, and THCA concentrations within the PLBO and CTC groups were not different than the associated LLOQ values. The authors believe compound interference or inadvertent sample contamination may be responsible for the CBDA, 9-THC, and THCA findings, as opposed to being a true positive. This theory is supported by the fact that concentrations of these cannabinoids were similar or higher in the PLBO and CTC steers compared to those receiving IH.

While the 9-THC, THCA, and CBDA findings are likely false positives, we believe the CBD-7-acid findings in the PLBO and CTC steers are reliable due to the precedent set by previous literature and the consistently detectable concentrations throughout the feeding period. Transference of residues in urine or feces to unexposed animals has been reported for non-steroidal anti-inflammatory drugs in pigs and horses (Popot et al., 2007; Hairgrove et al., 2019; Bates et al., 2020). Similar to human literature on disposition of cannabinoids (Grotenhermen, 2003), previous literature in cattle receiving IH has reported high levels of fecal and urine excretion (Addo et al., 2023; Smith et al., 2023; Fruge et al., 2025; Irawan et al., 2025). Specifically, Fruge et al. (2025) reported large standard deviations in their fecal cannabinoid measurements, indicating differences in individual animal’s cannabinoid metabolism. The authors believe that environmental contamination and residue transfer is the most plausible reason for consistent detection of CBD-7-acid in the PLBO and CTC steers. It is possible that residue transference was also responsible for the detectable CBDA, 9-THC, and THCA concentrations. However, the authors believe this situation is less likely than the compound interference or sample contamination scenarios, since there were no other samples with 9-THC concentrations above the LLOQ and no other CTC or PLBO samples with detectable THCA concentrations.

Based on the significant treatment effect for CBD-7-acid, it appears that concurrent administration of CTC may increase CBD-7-acid concentrations, potentially due to changes in cytochrome P450 (CYP) isoform levels or activity. The main CYP isoenzymes critical to CBD metabolism in humans are CYP3A4 and CYP2C19 (Jiang et al., 2011; Gaston and Friedman, 2017). While the role of CYP in CBD metabolism in bovines has not been established, four bovine CYP3A isoenzymes have been previously described, some of which correlate to human CYP3A4 (Zancanella et al., 2010). Furthermore, tetracycline has been shown to inhibit human CYP3A4 *in vitro* (Zhao and Ishizaki, 1997) and a study in pigs reported variable risk of CYP2 and CYP3 inhibition by oxytetracycline, chlortetracycline, and doxycycline (Hu et al., 2016). Future work should confirm our findings and investigate potential risks of coadministration of veterinary drugs and IH in cattle.

Although we detected CBD-7-acid up to 14 d after final administration, we cannot accurately estimate half-life from our results. Determining the half-life and other pharmacokinetic parameters would enable the application of CBD-7-acid for exposure time estimation. Development of a rapid analytical method for this cannabinoid could provide a useful tool for quickly identifying exposed cattle for further testing, as has been previously proposed by Xu and Murphy (2024). However, caution is warranted if an analytical method is developed for this purpose. The present study consistently identified CBD-7-acid in cattle not receiving IH, possibly secondary to exposure to urine and feces from cattle in the HEMP or COMBO groups. Thus, any future surveillance strategy should employ either a second, confirmatory test or should incorporate cutoff values that would exclude animals with detectable concentrations due to environmental contamination from exposed animals or should recommend confirmatory testing prior to any regulatory actions.

Limitations of this study include the small sample size and lack of follow-up past 14 d after final IH administration. There are no IH products approved in cattle and thus there is no tolerance for any cannabinoid in cattle tissues or plasma. If further regulatory discussions deem CBD-7-acid of importance for surveillance measures, additional studies should be performed with a longer follow-up period. Additionally, a relatively low dosage of cannabinoids was used in this study compared to previous feeding trials (Wagner et al., 2022; Irawan et al., 2025). Longer durations and higher inclusion levels than what was used in the present study may result in even more prolonged depletion of cannabinoids in plasma. The experimental vaccine confounding variable is another limitation. This effect was minimized, as we only compared the HEMP and COMBO groups, which both received the vaccine, and were merely reporting descriptive statistics for the CTC and PLBO groups. However, any differences in elimination between the HEMP and COMBO groups cannot be accurately ascribed to IH due to confounding vaccine status.

In the present study, we observed that acidic cannabinoids represented a majority of plasma cannabinoids. We detected the cannabinoid metabolite CBD-7-acid 14 d after final IH administration and believe that it could be a reliable indicator of prior IH exposure for use in future surveillance measures. These results will inform decisions regarding surveillance and interpretation of samples collected from cattle tested for cannabinoids or IH exposure.

## Supplementary Material

skaf418_Supplementary_Data

## Data Availability

All data used in preparation of this manuscript are in the main or supplementary files.

## Abbreviations:

8-THC: Δ8-tetrahydrocannabinol
9-THC: Δ9-tetrahydrocannabinol
CBC: cannabichromene
CBCA: cannabichromenic acid
CBG: cannabigerol
CBGA: cannabigerolic acid
CBD: cannabidiol (CBD)
CBD-7-acid: (−)-7-nor-7-carboxy cannabidiol
CBDA: cannabidiolic acid
CBDV: cannabidivarin
CBDVA: cannabidivarinic acid
CBLA: cannabicyclolic acid
CBN: cannabinol
CTC: chlortetracycline
FDA: United States Food and Drug Administration
IACUC: institutional animal care and use committee
IH: industrial hemp
LLOQ: lower limit of quantification
THC-11-OH: ( ±)-11-hydroxy-Δ9-tetrahydrocannabinol
THC-acid: (−)-11-nor-9-carboxy-Δ9-tetrahydrocannabinol
THC-acid-glu: (+)-11-nor-9-carboxy-Δ9-tetrahydrocannabinol glucuronide
THCA: Δ9-tetrahydrocannabinolic acid A
THCV: Δ9-tetrahydrocannabivarin
THCVA: Δ9-tetrahydrocannabivarinic acid
UPLC-MS: ultra-performance liquid chromatography triple quadrupole mass spectrometry
USDA: United States Department of Agriculture
