# Supplementary material for: Plasma cannabinoid concentrations and transference during long-term industrial hemp administration in cattle
Source: J Anim Sci. 2025 Dec 8;103:skaf418. doi: 10.1093/jas/skaf418 (PMC12770982; doi:10.1093/jas/skaf418)
Supplement: skaf418_Supplementary_Data [file skaf418_supplementary_data.zip › Long term hemp PK_Suppl cover and fig.docx]

Long-term plasma cannabinoids in cattle

**Plasma cannabinoid concentrations and transference during long-term industrial hemp administration in cattle^[[1]](#footnote-1)^**

SUPPLEMENTAL FILES

Bailey R. Fritz^*^, Michael D. Kleinhenz^†**^, Jason J. Griffin^‡^, Mikaela M. Weeder^*^, Alyssa A. Nelson^*^, Andrew K. Curtis^*||^, Geraldine Magnin^*§^, Jonathan Ferm^§#^, Roman R. Ganta^§#^, Johann F. Coetzee^*¶^

^*^Department of Anatomy & Physiology, College of Veterinary Medicine, Kansas State University, Manhattan, KS 66506 USA

^†^Department of Clinical Sciences, College of Veterinary Medicine, Kansas State University, Manhattan, KS 66506 USA

^‡^John C. Pair Horticulture Center, Kansas State University, Haysville, KS 67060 USA

^§^Department of Diagnostic Medicine/Pathobiology, College of Veterinary Medicine, Kansas State University, Manhattan, KS 66506 USA

^||^Current address: College of Veterinary Medicine, University of Missouri, Columbia, MO, USA 65201 USA

^#^Current address: Christopher S. Bond Life Sciences Center, University of Missouri, Columbia, MO 65201 USA

^¶^Current address: Office of the Vice President for Research, Kansas State University, Manhattan, KS 66506 USA

**Supplemental Fig. S1.** Concentrations of CBD-7-acid, CBDA, 9-THC, and THCA (back transformed logarithmic mean ± SE) in Holstein steers administered alfalfa pellet placebo (PLBO) or chlortetracycline (CTC; 1.1 mg/kg/d) by mouth once daily for 63 days. Sampling occurred every 7 days from day -7 to 77. No cannabinoids were detected on days -7, 0, or 7. Error bars are not shown if smaller than the symbol.


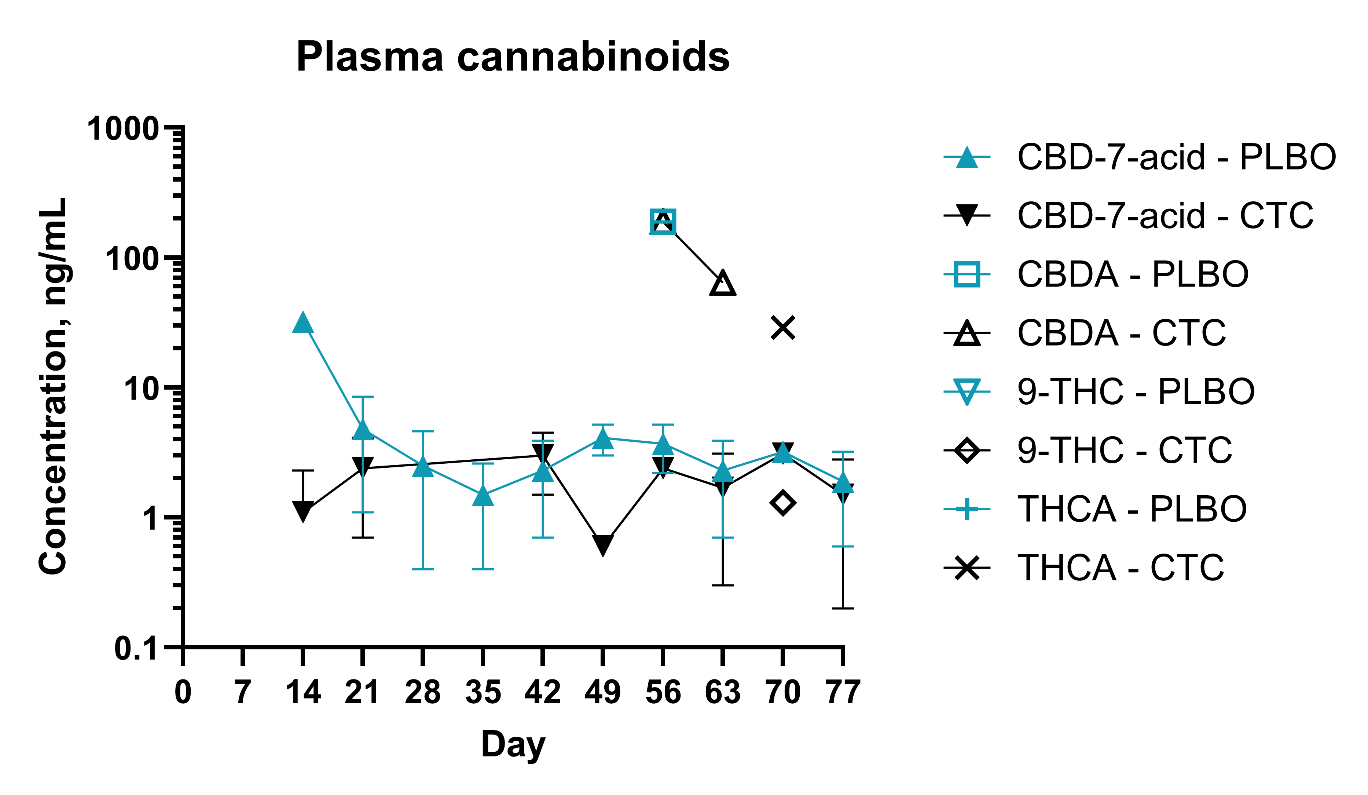


1. This work was supported by the Kansas State University College of Veterinary Medicine Office of Research and by the Agriculture and Food Research Initiative project award no. 2020-67030-31479, from the U.S. Department of Agriculture’s National Institute of Food and Agriculture. [↑](#footnote-ref-1)
